# Wisdom in Oncology: Balancing Cure and Quality of Life in Head and Neck Cancer

**DOI:** 10.7759/cureus.103006

**Published:** 2026-02-05

**Authors:** Adeel Riaz, Abu Hurrairah, Mariam Arif

**Affiliations:** 1 General Surgery, The Brooklyn Hospital Center, Brooklyn, USA; 2 Radiation Oncology, Rutgers Robert Wood Johnson Medical School, New Brunswick, USA; 3 Radiology, Aziz Fatima Medical and Dental College, Faisalabad, PAK; 4 Internal Medicine, Lincoln Medical and Mental Health Center, Bronx, USA

**Keywords:** clinical judgement, cure vs quality of life, de-escalation, head and neck cancer, long-term impacts of cancer treatment

## Abstract

This commentary reflects on the evolution of clinical judgment in oncology, arguing that mature practice requires not only knowing how to treat aggressively, but also when to refrain. It uses head and neck oncology - particularly HPV-positive oropharyngeal cancer - as a paradigm in which long-term toxicities such as dysphagia, xerostomia, fibrosis, pain, and dependence on nutritional support can transform cure into a life constrained by potentially avoidable morbidity. Highlighting de-escalation strategies tailored to biologic risk, and the growing emphasis on quality of life alongside survival, it calls for a shift from reflexive maximalism toward patient-centered restraint, where functional preservation and dignified survivorship are valued as highly as oncologic control.

## Editorial

Modern oncology has been shaped by decades of progress defined largely by survival gains. Advances in surgery, radiation, systemic therapy, and supportive care evidenced by landmark trials have shifted many cancers from fatal diagnoses to curable or chronic diseases. Yet this success has introduced a new challenge that becomes clearer only with time: survival alone is an incomplete measure of success. As the population of long-term cancer survivors grows, clinicians are increasingly confronted with the lived consequences of treatment - consequence of how patients live after treatment, not merely whether they live. This demands a more nuanced definition of what it means to heal. 

In the evolving landscape of oncology, few reflections capture the maturation of clinical judgment as poignantly as the observation that true wisdom lies not only in knowing how to treat aggressively, but in knowing when not to. Early in one’s career, the drive to pursue cure at all costs is understandable; a reflection of training emphasizing maximal intervention to eradicate disease. Yet, with experience comes the sobering recognition of treatment’s double-edged sword, patients rendered cancer-free but burdened by chronic morbidities that erode their independence, dignity, and daily joy.

This tension comes up in all areas of oncology but is starkly evident in head and neck oncology, where the triumph of survival often comes at great personal cost. Following definitive treatment - such as trimodality therapy (surgery, radiation, and chemotherapy), surgery followed by radiation only, or chemoradiation, or radiation alone - chronic dysphagia, xerostomia, fibrosis, pain, and dependence on nutritional support are not rare complications. These conditions can profoundly impair swallowing, speech, and social functioning, transforming survival into a life shadowed by possible avoidable toxicity. As clinicians accumulate years of follow-up data, they witness the “other side of success”, long-term survivors whose quality of life is compromised by the very interventions that saved them.

What distinguishes experience from inexperience is not diminished commitment to cure, but an expanded awareness of consequence. The oncologist’s responsibility extends beyond eradicating disease to anticipating the downstream effects of treatment decisions - effects that may unfold over decades rather than months. Follow-up clinics, survivorship visits, and patient narratives gradually recalibrate clinical instincts, replacing reflexive escalation with thoughtful deliberation. 

The responsibility of the oncologist extends beyond oncologic control to a nuanced balancing act: tailoring treatment intensity to the true biologic risk of the disease. This shift toward patient-centered care is increasingly supported by evidence. In HPV-positive oropharyngeal cancers, which now predominate and carry a favorable prognosis, standard regimens derived from historical HPV-negative cohorts may represent overtreatment. De-escalation trials are exploring reduced radiation doses, reduced radiation volumes, replacing, reducing or omitting chemotherapy, minimally invasive surgery with de-escalated (chemo)radiotherapy to preserve function without compromising cure rates [1-4]. Emerging data highlight that aggressive approaches, while effective for survival, often incur significant late morbidity, underscoring the need for restraint in low-risk subsets.

Importantly, restraint is not synonymous with undertreatment, nor does it reflect therapeutic nihilism. Rather, it represents precision, that is matching treatment intensity to disease biology and patient priorities. This precision must also extend to communication. Patients frequently equate more treatment with better care, a belief reinforced by cultural narratives of “fighting” cancer. Experienced clinicians learn that part of their role is reframing this narrative, helping patients understand that less intensive therapy may offer not less hope, but a better life.

Broader oncology literature reinforces this evolution. Reviews emphasize integrating quality-of-life metrics into regulatory approvals and clinical decisions, recognizing that survival alone is an incomplete endpoint [5]. Wisdom emerges from experience: prioritizing functional preservation alongside cure, weighing long-term impacts, and resisting the inertia of maximalism.

This evolution raises important implications for training and culture within oncology. Early career physicians are taught to act decisively and aggressively, often before they have witnessed the late effects of treatment. Incorporating survivorship, late toxicity, and longitudinal patient outcomes into education may help foster discernment earlier, narrowing the gap between technical expertise and clinical wisdom.

As the field advances with precision medicine, immunotherapy, and biomarker-driven strategies, the imperative grows clearer. Cure remains paramount, but not at the expense of a life diminished by toxicity. The mature oncologist’s greatest skill is discernment: deploying aggression when necessary and embracing restraint when it best serves the patient. In this balance lies not just better outcomes, but deeper humanity in cancer care.
